# Vitamin Pharmacogenomics: New Insight into Individual Differences in Diseases and Drug Responses

**DOI:** 10.1016/j.gpb.2016.10.005

**Published:** 2017-04-01

**Authors:** Hai-Yan He, Mou-Ze Liu, Yue-Li Zhang, Wei Zhang

**Affiliations:** 1 Department of Clinical Pharmacology, Xiangya Hospital, Central South University, Changsha 410008, China; 2 Institute of Clinical Pharmacology, Central South University, Hunan Key Laboratory of Pharmacogenetics, Changsha 410078, China; 3 International Medical Department, Xiangya Hospital, Central South University, Changsha 410008, China; 4 National Clinical Research Center for Geriatrics, Xiangya Hospital, Central South University, Changsha 410008, China

**Keywords:** Vitamin, Pharmacogenomics, Single nucleotide polymorphism, Genetic variation, Individual difference

## Abstract

Vitamins are vital to sustain normal physiological function, metabolism, and growth for all living organisms. Being an integral component of coenzyme, vitamins can affect the catalytic activities of many enzymes and the expression of drug transporters. Genetic variations in metabolism and/or transporter genes of drugs can influence the exposure of the human body to drugs and/or their active metabolites, thus contributing to the variations in drug responses and toxicities. Nonetheless, **pharmacogenomics** studies on nutrients have been rarely summarized. In this article, we reviewed recent progress on **vitamin** pharmacogenomics, for a better understanding on the influence of vitamin-related gene polymorphisms on inter-individual differences in diseases and drug efficacy and safety.

## Introduction

Pharmacogenomics (PGx), in a broad sense, refers to the study of DNA or RNA variations and their relationship with drug responses for better understanding of inter-individual differences in drug efficacy or safety. Examples include the association of genetic polymorphisms in the genes encoding the thiopurine methyltransferase (*TPMT*) with 6-mercaptopurine and azathioprine, genetic variation in the genes encoding cytochrome P450 2C9 (*CYP2C9*) and Vitamin K epoxide reductase complex subunit 1 (*VKORC1*) with warfarin [1], [2], and genetic variation in the genes encoding human leukocyte antigen B (HLA-B*5701) with abacavir [3]. They all demonstrate the importance of dose adjustments in order to guarantee the safety and effectiveness of drug therapy, or to identify high risk patients. In recent years, genetic tests have been increasingly performed in the treatment of cancer [4], cardiovascular disease [5], epilepsy [6], and HIV infection [7] to make drug therapy more effective and safer.

Pharmacokinetics (PK) and pharmacodynamics (PD) are influenced by genetic variations in genes encoding metabolic enzymes, membrane transporters, and/or receptors. Even when the results of a PGx assessment are neutral (no genomic effect is found), PGx can also streamline drug development by confirming that certain suspected pathways are not likely to contribute significantly to inter-individual variability in PK, PD, efficacy, or safety [8]. However, PGx studies on nutrients including carbohydrate, protein, fat, vitamin, mineral, and water are rare. In this review, we summarized recent progress on PGx of vitamins.

Vitamins are required for normal human physiology and some vitamins even participate in a variety of processes within the brain. The physiological roles of vitamins have been extensively investigated and their involvement in the regulation of nervous system dysfunction has also been reported recently [9]. Despite different chemical structures and characteristics, different vitamins share some common features. (1) Most vitamins in nature are synthesized from amino acids as precursors [10]. (2) Vitamins, whose key function is metabolism regulation, are neither components of cell, nor do they produce energy. (3) Except for Vitamin D, most vitamins cannot be synthesized nor produced in a sufficient amount by human bodies and thus usually should be supplemented by food. (4) Human body needs only a small amount of vitamins measured as milligrams or micrograms [11].

Although the quantity of vitamins demanded is very small, they are indispensable for humans to maintain normal physiological functions. Vitamin deficiency would pose serious threat to human health. For example, the deficiency of vitamin A may lead to night blindness [12], whereas vitamin D deficiency may cause rickets [13]. However, vitamins can’t exert their functions in the human body without a variety of enzymes, transporters, receptors, and binding proteins that are involved in the absorption, distribution, metabolism, and excretion (ADME) of vitamins. If polymorphisms occur in the genes encoding the aforementioned proteins, the *in vivo* processing and the corresponding function of vitamins can become aberrant, thus affecting disease treatment [14].

Vitamins are roughly classified into two types, *i.e.*, water-soluble vitamins and fat-soluble vitamins. The water-soluble vitamins do not require digestion, they can be directly absorbed in intestines and reach the required tissues via circulation. Fat-soluble vitamins can be dissolved in oil or fat. They are emulsified by the bile, absorbed in the small intestine, and delivered to different organs through lymphatic circulatory system.

## Vitamin D

**Figure 1:**
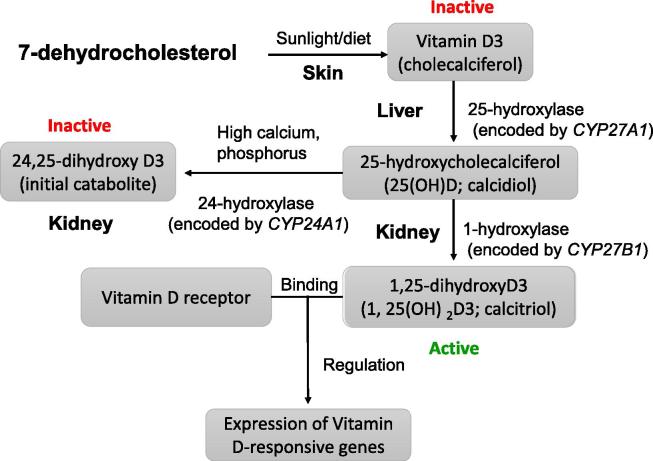
**The route of Vitamin D from synthesis to gene regulation** Vitamin D can be obtained from diet or skin exposure to sunlight. It is converted by 25-hydroxylase to calcidiol in the liver, which is then converted to calcitriol by 1-hydroxylase in the kidney to become biologically active. Calcitriol subsequently reaches target cells through circulation and binds to vitamin D receptor and elicits the expression of vitamin D-responsive genes. On other hand, calcitriol can be catalyzed by 24-hydroxylase to become inactive initial catabolite 24, 25-dihydroxy D3.

Vitamin D is one of the secosteroid hormones, which can be obtained from diet or sun exposure of skin. It functions by regulating intestinal calcium absorption [15]. After binding to the vitamin D binding protein (VDBP), vitamin D then can be transported to the liver and subsequently converted by 25-hydroxylase that is encoded by *CYP27A1* into the prohormone calcidiol, which is also known as 25-hydroxycholecalciferol or 25-hydroxyvitamin D [25(OH)D] [16] (Figure 1). Vitamin D is usually present in several different forms, among which D_2_ (ergocalciferol) and D_3_ (cholecalciferol) are two major indispensable forms. Vitamin D_2_ is mainly produced in natural plants, whereas vitamin D_3_ is primarily synthesized from 7-dehydrocholesterol in the skin via sun exposure and converted by 1α-hydroxylase into the biologically-active calcitriol (1,25-dihydroxy cholecalciferol or 1,25-dihydroxyvitamin D_3_; 1,25(OH)_2_ D_3_) in the kidney. About 95% of vitamin D in human body is produced in the skin through sunlight-dependent processes [17], [18]. The regulation of vitamin D production process is strongly dependent on renal 1α -hydroxylase. Expression of renal 1α-hydroxylase can be up-regulated by parathyroid hormone (PTH) and inhibited by calcitriol itself. Then in the kidneys, both calcidiol and calcitriol will be converted by 24-hydroxylase into calcitroic acid, a water-soluble inactive compound, which is excreted into bile [16].

1α -hydroxylase and 24-hydroxylase, which are encoded by *CYP27B1* and *CYP24A1* in humans, respectively, are of critical importance in governing calcitriol concentrations. It has been proposed that these two enzymes work jointly to regulate the concentration of calcitriol at the tissue level. Genetic polymorphisms in both *CYP27B1*and *CYP24A1* (Table 1) have been identified, which show significant association with the concentrations of vitamin D metabolites in circulation and with the risk of colorectal cancer [19], [20]. Vitamin D deficiencies may lead to declined cognitive function, dementia, and Alzheimer's disease [21].

Vitamin D-related endocrine dysfunction may contribute to autoimmune thyroid disease (AITD), such as systemic sclerosis (SSc) and rheumatoid arthritis (RA) [22]. It was once reported by Pani et al. that the variations in intron 8 (TAAA) n variable tandem repeats of *DBP* that encodes VDBP, the main transporter for calcitriol endocytosis, were significantly correlated with Graves' disease [23]. In a cross-sectional study including 56 men with idiopathic osteoporosis as well as 114 healthy controls, Al-oanzi et al. investigated variations in (TAAA) n-Alu of *DBP*. They found that DBP-Alu*10 and *11 alleles showed protection effect on patients with osteoporosis disease (OR = 0.39 and OR = 0.09, respectively). Carriers with 19–20 repeats (genotype 9/10, 9/11, and 10/10) had higher concentration of VDBP and vitamin D in circulation, higher bone mineral density, and reduced risk for developing osteoporosis [24].

The binding of calcitriol to the vitamin D receptor (VDR) is required before it regulates the expression of vitamin D-responsive genes. Levin et al. examined 141 SNPs in 1514 white participants from the United States and found that an intergenic SNP (rs7968585) located 3.2 kb downstream from *VDR* was associated with risk of the composite outcome (incident hip fracture, myocardial infarction, cancer, and mortality over long-term follow-up). Subjects carrying one minor allele presented hazard ratios of 1.4 (95% CI, 1.12–1.74) for composite outcome [25].

## Vitamin E

The fat-soluble vitamin E is known to be made up from tocopherols and tocotrienols. Vitamin E has prevention effects on the oxidation of phospholipids and polyunsaturated fatty acids, helps to maintain integrity of cell membrane, reduces blood lipid peroxide, prevents platelet aggregation, increases the stability of erythrocyte membrane, promotes the synthesis of red blood cells, *etc*. [26]. Previous studies have discovered that α-tocopherol (α-T) is one of the most biologically active forms of vitamin E and a lipid-soluble antioxidant [9], [27]. After hepatic uptake, α-T can be resecreted by α-T transfer protein (α-TTP) from liver to plasma [28]. The genetic polymorphism of *a-TTP* can affect functions of vitamin E.

The transportation of vitamin E relies on triglyceride-rich lipoprotein (TRL) *in vivo*. Its tissue distribution may be driven by lipoproteins: low density lipoprotein (LDL) transfers vitamin E to tissues, whereas high-density lipoprotein (HDL) participates in reversing the transportation of vitamin E, *i.e.*, transferring it from tissues back into the liver [29]. Apolipoprotein A5, which is encoded by *APOA5*, plays an important role in regulating the plasma triglyceride levels. Polymorphism in *APOA5* is significantly associated with triglyceride (TG) concentration in the plasma, suggesting that vitamin E transportation may be regulated by APOA5 *in vivo* [30]. In type 2 diabetes patients with *APOA5* 1131T>C heterozygous mutation, the vitamin E level was about 13% higher than normal population. The probability of patients with higher vitamin E levels in TC genotype was about 2.6 times of that in normal population [30], [31].

Vitamin E can effectively lower the levels of plasminogen activator inhibitor 1 (PAI-1), an inhibitor of fibrinolysis. PAI-1 is encoded by *SERPINE1* in humans and known as one of the risk factors to the cardiovascular diseases. The expression of PAI-1 can be influenced by *4G/5G* polymorphism of *SERPINE1* [32]. For a cohort containing 93 type II diabetic patients that were given a daily dose of 500 IU vitamin E for 10 consecutive weeks, Testa et al. found that, PAI-1 expression levels began to drop from the 10th week in patients carrying *4G/4G* or *4G/5G* genotype, while PAI-1 expression levels began to drop from the 5th week in patients with *5G/5G* genotype (*P* < 0.01). These findings indicated patients with 5G/5G genotype may benefit more from vitamin E treatment for cardiovascular disease prevention [32].

## Vitamin K

Vitamin K is also called the clotting vitamin, because it can promote blood clotting. Additionally, it also is involved in anticalcification, anticancer, bone formation, and insulin sensitization [33]. Vitamin K is known to be significantly related with the bone mineral density of elderly people [34]. There are two major forms of vitamin K: vitamin K_1_ and vitamin K_2_, with the former synthesized by natural plants and the latter by microorganisms. Human intestinal bacteria can also synthesize vitamin K_2_. The vitamin K intestinal absorption process includes bile-dependent solubilization, uptake into the enterocytes, packaging, and exocytosis to the lymphatic system, which is similar to that for the lipids.

*APOE* genetic polymorphisms are known to affect transportation and cellular uptake of lipoprotein. Plasma vitamin K level in hemodialysis patients was found to be correlated with the genotype of *APOE* (order E2 > E3 > E4) [35]. In another study on Chinese and UK healthy elders, researchers found that the vitamin K1 level in individuals with E4/4 or E3/4 genotype was significantly higher than those with E3/3 or E2/3 genotype, while the percentage of undercarboxylated osteocalcin (ucOC; total osteocalcin adjusted) was lower in *APOE4* allele carriers than people with other genotypes in Chinese [36]. Kohlmeier et al. reported that *APOE* genotype could influence vitamin K levels in the blood, which might be a significant determinant of bone fracture [34].

The vitamin K-epoxide cycle is very important to the function of vitamin K and its storage in the microsomal cells. Two major integral membrane proteins participate in the process: g-glutamic carboxylase (GGCX) and vitamin K epoxide reductase (*VKOR*, both of which are suggested to be involved in reduction and oxidation of membrane-bound vitamin K [37]. Polymorphisms in *VKORC1* and *GGCX* can influence the vitamin K recycle in liver, which might hold true in the extra-hepatic tissues as well [38]. There is evidence suggesting that common polymorphisms of genes involved in vitamin K metabolism (*e.g.*, *APOE* and *VKOR*) might lead to abnormal levels of vitamin K, making dose adjustment necessary. However, the observed genetic effects need to be validated by case−control studies with large sample sizes.

## Vitamin B_12_

Vitamin B_12_, also known as coalmine due to the inclusion of cobalt, is the only vitamin that contains metal element. Vitamin B_12_ can be synthesized by intestinal microorganisms or obtained from diet. Three key proteins, haptocorrin (HC), intrinsic factor (IF), and transcobalamin II (TCII), are responsible for the pharmacokinetic absorption and cellular uptake of vitamin B_12_ [39]. Vitamin B_12_ participates in erythrocyte formation and DNA synthesis. Therefore, long-term inadequate intake of vitamin B_12_ may eventually lead to hematological disorders or other clinical disorders such as body immune deficiency, megaloblastic anemia, coronary heart disease, gastrointestinal, and nervous system diseases [40]. Studies in Indian population have reported that a low level of vitamin B_12_ was related to a higher risk of coronary artery disease [41], [42]_._

In recent years, identification of diseases-related SNPs has been greatly facilitated by the application of high-density genotyping arrays and genome-wide association studies (GWAS). Some SNPs that are reported to influence vitamin B_12_ levels have been identified. For instance, SNP 772G>A (rs602662) in the exon 2 of the gene encoding fucosyl transferase (*FUT2*) was related with the alterations in plasma vitamin B_12_ levels [43]. Notably, rs602662 has been repeatedly identified by GWAS analyses. Compared to individuals with AA or GA genotypes, individuals with GG genotype possessed the lowest levels of vitamin B_12_ (median; 175.3, 152.7, and 149.5 pM for AA, GA and GG, respectively). Furthermore, vegetarians with GG phenotype had a significantly lower plasma vitamin B_12_ level than non-vegetarians with AA genotype (140.7 pM *vs.* 174.2 pM) [44].

Vitamin B_12_ exists in the form of methylcobalamin and acts as a coenzyme of methionine synthase that catalyzes the methyl group transfer from 5-methyl-tetrahydrofolate. Polymorphisms in genes encoding transfer proteins of vitamin B_12_ may influence the cellular uptake process of vitamin B_12_ complex. Among them, 776C>G (rs1801198) in the gene encoding transcobalamin (*TCN2*), which leads to the substitution of arginine with proline, is the most common polymorphism [45]. This polymorphism may affect the binding affinity of TC to vitamin B_12_ and its ability of transporting vitamin B_12_ into tissues (Table 2). Individuals with *TCN2* 776GG genotype had significantly lower levels of HC−vitamin B_12_ complex, lower percentage of total transcobalamin-vitamin B_12_, but higher concentration of plasma methylmalonic acid (MMA). These findings suggest that the *TCN2* 776C>G polymorphism may influence the availability of vitamin B_12_, thus leading to a low vitamin B_12_ status. In a cohort containing 359 young healthy non-pregnant women, Von et al. reported significantly lower levels of transcobalamin-vitamin B_12_ complex in the plasma of individuals with 776GG genotype than those with 776CC genotype (74 *vs.* 87 pM; *P* = 0.02), suggesting less vitamin B_12_ available for cellular uptake and metabolism in the former individuals [45]. Future investigations are needed to verify whether there would be functional effects on diseases when the *TCN2* 776C>G polymorphism is coupled with inadequate dietary vitamin B_12_ intake.

## Vitamin C

Vitamin C is a water-soluble vitamin, also known as L-ascorbic acid, which is a very important scavenger for the endogenous free radical and can be neuroprotective by reducing damages from excito-toxicity [9]. The main function of vitamin C is to help human body to accomplish the REDOX reaction, thus important in preventing diseases, such as cancer, atherosclerosis, and rheumatism, as well as in enhancing immunity. Vitamin C has been considered to have no harm, since the kidney can drain off excessive vitamin C. It should be noted that vitamin C is essential to many biosynthetic processes that sufficient uptake is required for everybody. For example, vitamin C can improve endothelial function that is involved in cardio protection and collagen synthesis. Deficiency of vitamin C may lead to decreased collagen in atherosclerotic plaques, thus causing plaque rupture, blood clots, and even death in severe cases [46].

Sodium-dependent vitamin C transporters (SVCTs) are responsible for intake and discharge of ascorbic acid to maintain the homeostasis of vitamin C *in vivo*. Among them, SVCT2 functions to ensure the intracellular accumulation of ascorbic acid against the concentration gradients in the aorta or other active metabolism tissues [47]. The intronic SNP rs6139591 C>T in *SVCT2* (also known as *SLC23A2*) (Table 2) is associated with the alterations in intake of vitamin C and concentration of circulating ascorbic acid. A large-scale clinical cohort study involving 57,053 cases followed-up for 6.4 years reported that the absorption of vitamin C from food is less in the female patients carrying rs6139591 TT genotypes. These patients had a higher risk (increased 5.39-fold) of acute coronary syndrome than patients carrying rs6139591 *CC* genotypes [47]. In addition, women with homozygous rs1776964 TT genotypes of *SVCT2* can absorb more vitamin C than carriers of *CC* genotype. These results indicate that polymorphisms of the *SVCT2* gene may be related to risk of acute coronary syndrome in females [46], [47]. Therefore, vitamin C supplement may be effective for preventing acute coronary syndrome in females.

## Folic acid

Folate or folic acid (FA) belongs to vitamin B9, which is water-soluble and can be obtained from natural food or dietary supplements. FA, as well as the cobalamin and pyridoxine, is one of the key cofactors in homocysteine cycle, which are crucial for the synthesis of norepinepherine, creatine, melatonin, and even DNA [48]. Folate is also important in protein biosynthesis and central to cell viability.

Methylenetetrahydrofolate reductase (MTHFR) is the major enzyme for folic acid metabolism *in vivo*. Meta-analysis on genetic polymorphism 677C>T in *MTHFR* showed that people with TT genotype have a higher risk (CI: 14%−21%) of suffering from cardiovascular diseases than C allele carriers [49]. In addition, Torre et al. have found that *MTHFR* 677C>T polymorphism can influence the occurrence (*P* = 0.037) of colorectal cancer in a folic acid-dependent manner. Patients with *MTHFR* 677TT genotype have a higher risk of colorectal cancer (OR = 2.4) when their FA levels are low [50].

The protein reduced folate carrier (RFC-1), which is encoded by *SLC19A1*, participates in FA transportation across the placenta and the blood–brain barrier. FA deficiency can lead to abnormal DNA methylation profiles, which may ultimately lead to congenital disorders, cardiovascular diseases neuropsychiatric diseases or even cancer [51]. *RFC-1* genetic polymorphism 80A>G is reported to be related with a decreased FA level and some kind of congenital diseases such as ischemic heart disease [52]. Coppede et al. performed a meta-analysis involving 930 mothers with Down syndrome (DS) children [52] and showed that *RFC-1* 80GG genotype carriers had 1.27 times higher risk to have DS children, while the risk of having DS children was increased by about 1.14-fold in patients carrying a single G allele. This finding suggests that genetic polymorphism of *RFC-1* may have effects on fetal neuronal development via regulating the transportation of FA.

## Challenges and perspectives

Most of the studies up till now have demonstrated a potential link between SNPs and the absorption, transportation, metabolism and excretion processes of vitamins. However, the conclusions could be controversial under the clinical conditions. In the clinic, the nutritional status observed may change under disease status. Basic nutrition supplements can significantly improve patients’ functional outcomes and living standard. However, the typically-formulated standard nutritional replacement for patients contains only carbohydrates, proteins, and fats, but no vitamin or other minerals included. Some symptoms of disease would be exacerbated under the condition of vitamin deficiencies, which in turn would be a barrier for the body respond to many other drug therapies. In recent years, pharmacogenomics studies on vitamins and other nutrition components have generated increasing interest due to their potential to be primary therapeutics. However, there still lacks strong evidence for gene−vitamin interactions. Many vitamin-related pharmacogenomics studies are flawed due to defects in experimental design, small sample size, or short observation period. Future studies are needed to confirm the pharmacogenomics effect of vitamins and their involvement in the related disease processes. Illustrating the exact mechanisms underlying the vitamin pharmacogenomics involvement in drug pharmacokinetics and pharmacodynamics will facilitate better understanding in variations of inter-individual drug responses. It is our hope that vitamin-related pharmacogenomics studies will yield more convincing data and will offer guidance for clinical applications in the coming precision medicine era.

## Competing interests

The authors have declared no competing interests.

## Figures and Tables

**Table 1:** Effect of genetic polymorphisms on fat-soluble vitamins

**Vitamin**	**Gene**	**Protein function**	**Polymorphism**	**Effect of genetic polymorphisms on vitamins**	**Disease associated with vitamin deficiency**	**Refs.**
D	*CYP27B1*	1-Hydroxylase; key enzyme in generating the biologically-active calcitriol	rs28934604 (R107H)	Remarkably reduced activity in converting calcidiol to calcitriol (55% of wild type)	Calcium and phosphorus absorption barriers; declined cognitive function, dementia, and Alzheimer's disease	[19], [20]

	*CYP24A1*	24-Hydroxylase; key enzyme in transforming calcidiol and calcitriol into the inactive form	rs6068812 (L409S)	Remarkably reduced activity in catabolizing calcitriol (31% of wild type)		

	*DBP*	Vitamin D binding protein; the main transporter for calcitriol endocytosis	*10 and *11	Protecting effect on patients with osteoporosis disease		[24]
	
	*VDR*	Vitamin D receptor	rs7968585	Increased hazard ratios for composite outcome (incident hip fracture, myocardial infarction, cancer, and mortality over long-term follow-up)		[25]
	
E	*APOA5*	Apolipoprotein A5; Participates in the transportation of lipoprotein	rs662799 (−1131T>C)	Higher vitamin E level	Reproductive disorders; muscle, liver, bone marrow and brain dysfunction; erythrocyte hemolysis	[31]
	
	*PAI-1*	Plasminogen activator inhibitor 1; Encodes a protein that functions as an inhibitor of fibrinolysis	4G/5G	Benefiting more from vitamin E treatment for cardiovascular disease prevention		[32]
	
K	*APOE*	Apolipoprotein E; Transport and cellular uptake of lipoprotein	E3/4 and E4/4	Significantly higher vitamin K1 levels	Easy bleeding, anemia, long blood coagulation time	[35], [36]

**Table 2:** Effect of genetic polymorphisms on water-soluble vitamins

**Vitamin**	**Gene**	**Protein function**	**Polymorphism**	**Effect of genetic polymorphisms on vitamins**	**Disease associated with vitamin deficiency or excess**	**Ref.**
B_12_	*FUT2*	Fucosyl transferase; transporter required for cellular uptake of B_12_	rs602662 (772G>A)	Low concentrations of cellular and plasma vitamin B_12_ in G allele carriers	Deficiency: pernicious anemia	[44]
	
	*TCN2*	Transcobalamin II; transfer protein of vitamin B_12_	rs1801198 (776C>G)	Significantly lower levels of haptocorrin–vitamin B_12_ complex, lower percentage of total transcobalamin–vitamin B_12_ in GG allele carriers		[45]
	
C	*SVCT2* (*SLC23A2*)	Sodium-dependent vitamin C transporters; transporter responsible for intake and discharge of ascorbic acid to maintain the homeostasis of vitamin C	rs6139591	Higher risk of acute coronary syndrome when taking lower median dietary vitamin C in TT allele carriers	Deficiency: scurvy and skin purpura; gum bleeding Excess: urinary stones	[47]

			rs1776964	More vitamin C absorption in TT allele carriers than carriers of CC genotype.		

Folic acid	*MTHFR*	Methylenetetrahydrofolate reductase; major enzyme for folic acid metabolism	rs1801133 (677C>T)	Higher risk of cardiovascular diseases in TT allele carriers than C allele carriers	Deficiency: giant young red blood cell anemia, leukopenia	[52]

	*SLC19A1* (*RFC-1*)	Solute carrier family 19 member 1; transporting folic acid across the placenta and the blood–brain barrier	rs1051266 (80A>G)	Higher risk to have Down syndrome children in GG allele carriers		
